# *KRAS* codon 12 mutations characterize a subset of de novo proliferating “metaplastic” Warthin tumors

**DOI:** 10.1007/s00428-023-03504-x

**Published:** 2023-02-08

**Authors:** Abbas Agaimy, Konstantinos Mantsopoulos, Heinrich Iro, Robert Stoehr

**Affiliations:** 1 Institute of Pathology, University Hospital Erlangen, Friedrich‐Alexander University Erlangen‐Nürnberg (FAU), Krankenhausstraße 8-10, 91054 Erlangen, Germany; 2 Department of Otorhinolaryngology, Head and Neck Surgery, University Hospital Erlangen, Friedrich‐Alexander University Erlangen‐Nürnberg (FAU), Erlangen, Germany

**Keywords:** Salivary glands, Warthin tumor, Cystadenolymphoma, Metaplastic Warthin tumor, Mucoepidermoid carcinoma, KRAS mutations

## Abstract

Warthin tumor (WT; synonym: cystadenolymphoma) represents one of the most frequent salivary gland tumors with a frequency equaling or even outnumbering that of pleomorphic adenomas in some series. Histologically, the tumor displays tall columnar oncocytic cells, arranged into two cell-thick layers lining variably cystic glands within an organoid lymphoid stroma. Tumors with exuberant squamous metaplasia in response to FNA-induced or other types of tissue injury/infarction have been referred to as “*metaplastic WTs*.” However, the same terminology was used for tumors with variable mucinous cell and solid or stratified epidermoid proliferations (occasionally mimicking mucoepidermoid carcinoma), although the “metaplasia concept” has never been proven for the latter. We herein investigated 22 WTs showing prominent mucoepidermoid-like or solid oncocytoma-like proliferations without prior FNA or histological evidence of infarction/ trauma using the TruSight Tumor 15 gene panel and *KRAS* pyrosequencing. As a control, we tested 11 conventional WTs. No statistically significant differences were observed between the two subcohorts regarding patient’s age and tumor size. Six of 22 (27%) proliferating/ metaplastic WTs revealed oncogenic *KRAS* mutations clustering at codon 12 (exon 2), while all conventional tumors lacked these mutations. Our findings are in line with a neoplastic nature of the epidermoid/ mucoepidermoid proliferations in non-injured “metaplastic” Warthin tumors. We propose the descriptive term “de novo proliferating Warthin tumor” for this variant to distinguish it from infarcted/inflamed genuine metaplastic Warthin tumor.

## Introduction

Warthin tumor (WT; synonym: cystadenolymphoma) is a benign epithelial tumor of the salivary glands that occurs almost exclusively in the parotid gland and its associated lymph nodes [1]. Up to 12–20% of lesions are multifocal, and 5–17% of them are bilateral [1]. The tumor mainly affects adults in their 6th to 7th decades of life with variable predilection for males and association with cigarette smoking [1]. The recently observed gradual correction of the striking historical predilection for males might be attributable to changes in smoking habits among females.

Histologically, WTs display a double-layered columnar oncocytic epithelium lining variably cystic tubular glands with papillary projections, surrounded by organoid lymphoid stroma that recapitulates the structure of normal lymph node [2, 3]. However, the morphology of WT varies strikingly regarding the epithelial and lymphoid component [2, 3]. Notably, the epithelial component varies from pauciglandular or oligocystic tumors rich in predominant lymphoid stroma to lymphocyte-poor solid lesions hardly distinguishable from oncocytoma [2, 3].

At the cytological level, presence of non-oncocytic cells, mainly a few scattered mucous cells, represents a common feature in otherwise typical WTs [1–3]. However, ciliated epithelium and sebaceous cells are uncommon [1–3]. Moreover, some lesions display prominent solid epidermoid cell component with or without mucous cells. This proliferative component may rarely predominate and obscure the underlying double-layer pattern of WTs and has been a source of diagnostic confusion [2, 3]. Historically, these variant WTs have been collectively referred to as “metaplastic WTs” and have not been separated from tumors showing florid metaplastic squamous proliferations in response to fine needle aspiration (FNA)-related or other type of ischemic or mechanical tissue injury/infarction [4].

In this study, we report for the first time oncogenic *KRAS* mutations in a subset of tumors in the spectrum of “de novo metaplastic Warthin tumors,” for which we herein propose the descriptive term “*de novo proliferating Warthin tumors*.”

## Material and methods

All cases were routine cases treated at our center. The presence of a variable non-oncocytic epidermoid component forming solid nests or larger stratified aggregates with or without accompanying goblet cell component was used as the defining feature of proliferating/metaplastic WTs in this study. One tumor showing transition from conventional WT to an oncocytoma-like solid component was included as well. Tumors with evidence of tissue injury (infarction or prior FNA) have been excluded from this study. Eleven conventional WTs that lacked atypical or metaplastic features were tested as a control group. The tissue specimen was fixed in formalin and processed routinely for histopathology. Immunohistochemistry (IHC) was performed on a subcohort of the atypical cases (*n* = 17) using 3-µm sections cut from paraffin blocks with a fully automated system (“Benchmark XT System”, Ventana Medical Systems Inc., 1910 Innovation Park Drive, Tucson, Arizona, USA) and antibodies against p40 (polyclonal, 1:100, Zytomed) and Ki67 (clone MiB1, 1:100, Dako).

### Molecular genetic analysis

For DNA extraction, the whole tumor area was marked in conventional cases. In the atypical proliferating (metaplastic) tumors, the metaplastic area was selected for DNA extraction. In a subset of cases (*n* = 5), the conventional WT and the atypical proliferating areas were tested separately. After manual microdissection of the tumor cells and DNA isolation (Maxwell 16 system, Promega, Madison, USA), amplicon-based massive parallel sequencing was performed using the commercially available TruSight Tumor 15 (TST15) Panel, Illumina, San Diego, USA, and a MiSeq system according to the manufacturer’s instructions (Illumina) as described previously [5]. The TST15 gene panel is focused on the detection of hot-spot mutations within the coding regions of 15 genes that are frequently altered by mutations in solid tumors (*AKT1*, *BRAF*, *EGFR*, *ERBB2*, *FOXL2*, *GNA11*, *GNAQ*, *KIT*, *KRAS*, *MET*, *NRAS*, *PDGFRA*, *PIK3CA*, *RET*, *TP53*). Raw sequencing data was automatically aligned to the human genome (hg19), and the reported variants were annotated using Variant Studio 3.0 (Illumina). To validate the next-generation sequencing (NGS), and to exclude a lower sensitivity of the panel used, the same cohort has been tested using a Pyrosequencing assay specific to *KRAS* codons 12 and 13 using the multiplex PCR-kit according to manufacturer’s instructions (Qiagen, Hilden, Germany) and the following primers: forward: 5′-GGCCTGCTGAAAATGACTG-3′, and reverse: 5′-biotin AGCTGTATCGTCAAGGCACTCT-3′. For pyrosequencing (PyroMark Q24; Qiagen), single-stranded DNA was prepared from 25 ml of biotinylated PCR product with streptavidin-coated Sepharose and 0.5 mM of the sequencing primer 5′-CTTGTGGTAGTTGGAGC-3′ using the PSQ Vacuum Prep Tool (Qiagen).

### FISH testing for *MAML2* rearrangements

Three representative tumors (cases 17–19 in Table 1) have been initially evaluated for *MAML2* rearrangement. In addition, 14 tumors were tested for the sake of the current study using the same Zyto*Light* SPEC *MAML2* Dual Color Break Apart FISH Probe designed for detection of translocations involving the human *MAML2* gene at 11q21 (retrieved from ZytoVision, Bremerhaven, Germany) with standard protocols according to the manufacturer’s instructions. A cutoff value of > 10% of nuclei showing clear-cut split signals was defined as positive.

**Table 1:**
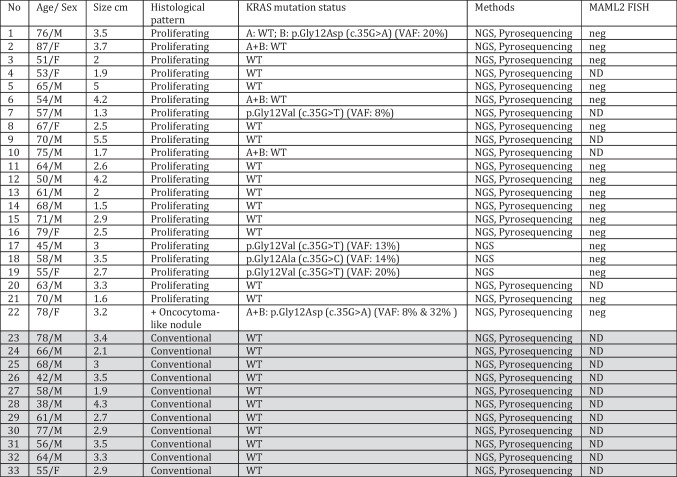
Clinicopathological and molecular findings in the whole study cohort of Warthin tumors (*n* = 33)

*A* conventional tumor component, *B* proliferating component, *ND* not done, *neg* negative, *NGS* next-generation sequencing using the TST15 panel, *WT* wild type, *VAF* variant allele frequency

*Conventional cases (Case 23 to 33) are shaded

## Results

### Demographic and clinical features

Clinicopathological features of the study cohorts are summarized in Tables 1 and 2. All tumors originated in the parotid gland. Patients were 15 males and 7 females in the metaplastic subcohort (M/F = 2.1/1) and 10 males and one female in the conventional type of tumor subcohort. The age range was 45 to 87 years (median, 64.5) and 38 to 78 (median, 61) for the metaplastic and the conventional type subcohort, respectively. Treatment was complete excision in all cases. No recurrences have been recorded in any of the cohorts during the follow-up period. None of the patients had a history of prior FNA.

**Table 2:** Compared clinicopathological features in conventional and proliferating Warthin tumors

Feature	Conventional Warthin tumors	Proliferating Warthin tumors
Age range (median)	38–78 (61)	45–87 (64.5)
Male/female ratio	10:1	2.1:1
Size range (median) in cm	1.9–4.3 (3)	1.3–5.5 (2.9)
*KRAS* mutations (%)	0/11 (0%)	6/22 (27%)

### Pathological findings of Warthin tumors

Tumor size ranged from 1.3 to 5.5 (median, 2.9) and 1.9 to 4.3 cm (median, 3) for the metaplastic and conventional tumors, respectively. Their cut-surfaces were described as grey whitish to tan and homogeneous with soft to firm consistency but varied greatly according to the presence and extent of the cystic component.

**Fig. 1:**
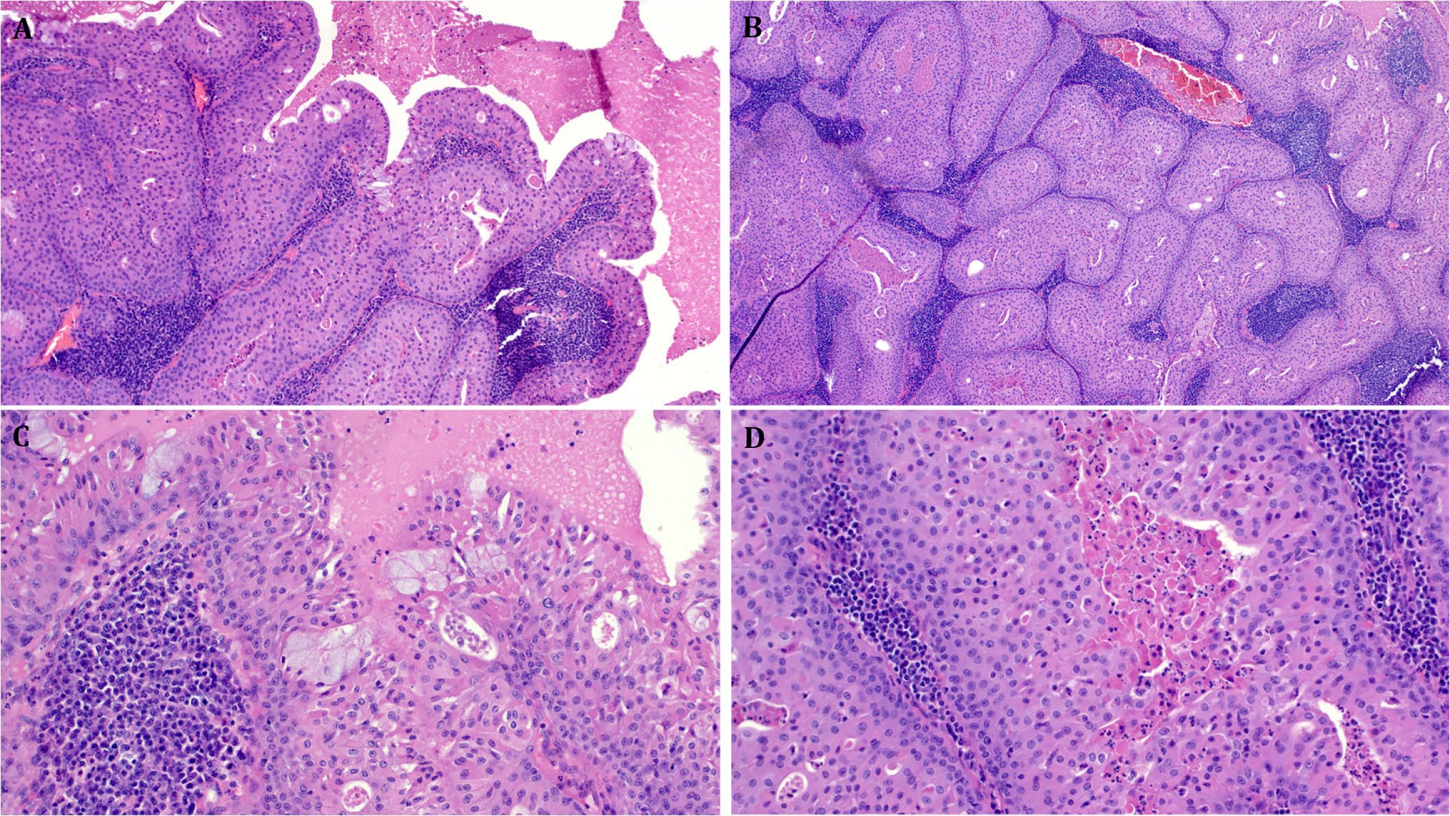
Representative images of de novo proliferating Warthin tumors. **A** This tumor shows classical papillary cystadenolymphoma pattern (right) merging with solid compact epidermoid proliferation (left) with a few scattered mucous cells. **B** Another area shows predominance of solid epidermoid proliferations, note residual small lumina and compressed and rarified, but unremarkable lymphoid stroma. **C** Higher magnification illustrating mucous cell clusters, note organoid arrangement of the stratified epithelium with basal cell hyperplasia and more oncocytic looking cells towards the surface. **D** Higher magnification of the epidermoid aggregates, not the Warthin-typical secretion in the residual lumen. All images from Case 17

**Fig. 2:**
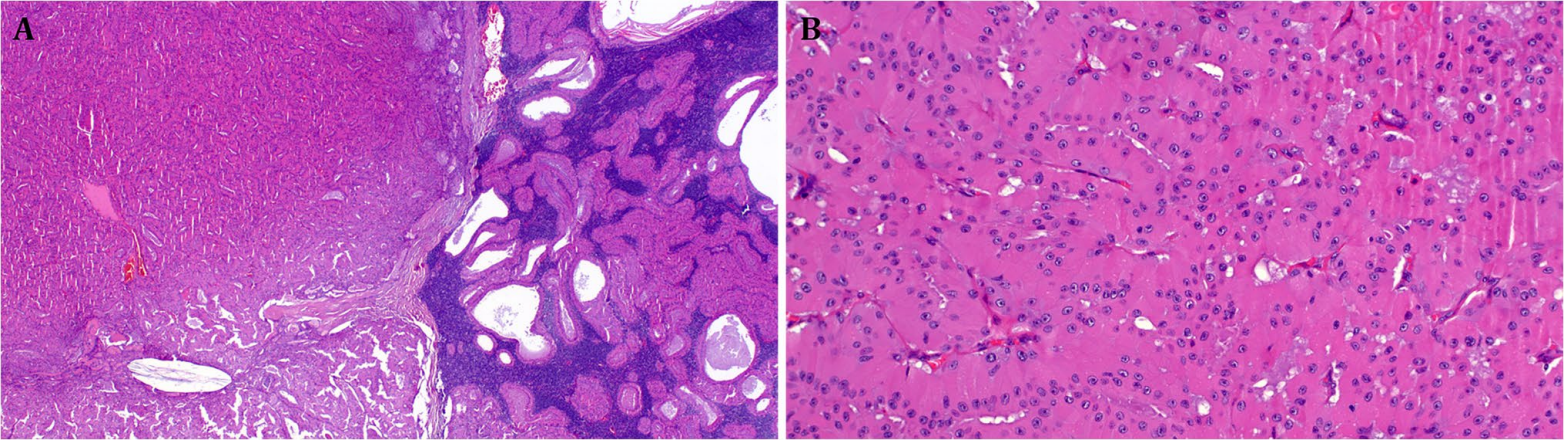
**A** This tumor showed transition from conventional Warthin tumor (right) to solid oncocytoma-like nodule devoid of lymphatic tissue (left). **B** Higher magnification of the solid oncocytoma-like nodule. Same *KRAS* mutation was detected in both components. All images from Case 22

Histologically, all conventional tumors showed a variable combination of tubules, cystic glands, and papillary projections lined or covered by tall columnar cells arranged into two layers supported by variable organoid lymphoid stroma. The metaplastic tumors revealed gradual transition from the conventional component to areas composed of variable monomorphic bland epidermoid cells arranged into several compact layers or solid aggregates interrupted by variable oncocytic or goblet cell elements, comprising between 10 and > 50% of the tumor (Fig. 1). One tumor revealed transition from conventional WT to a solid oncocytoma-like nodule almost devoid of lymphocytes (Fig. 2). None of the tumors revealed frankly squamous cells or basophilic atypical regenerative squamous and basal cell proliferations as seen in reparative squamous metaplasia characteristic of injured or infarcted WTs. The stroma in these metaplastic areas was compressed by the solid aggregates and represented by minimal residual lymphoid cells. Notably, no foci of necrosis or infarction, stromal fibrosclerosis, scarring, old and fresh hemorrhages, foamy histiocytic aggregates, granulocytes, or granulomatous reaction were seen. Mitoses were absent.

**Fig. 3:**
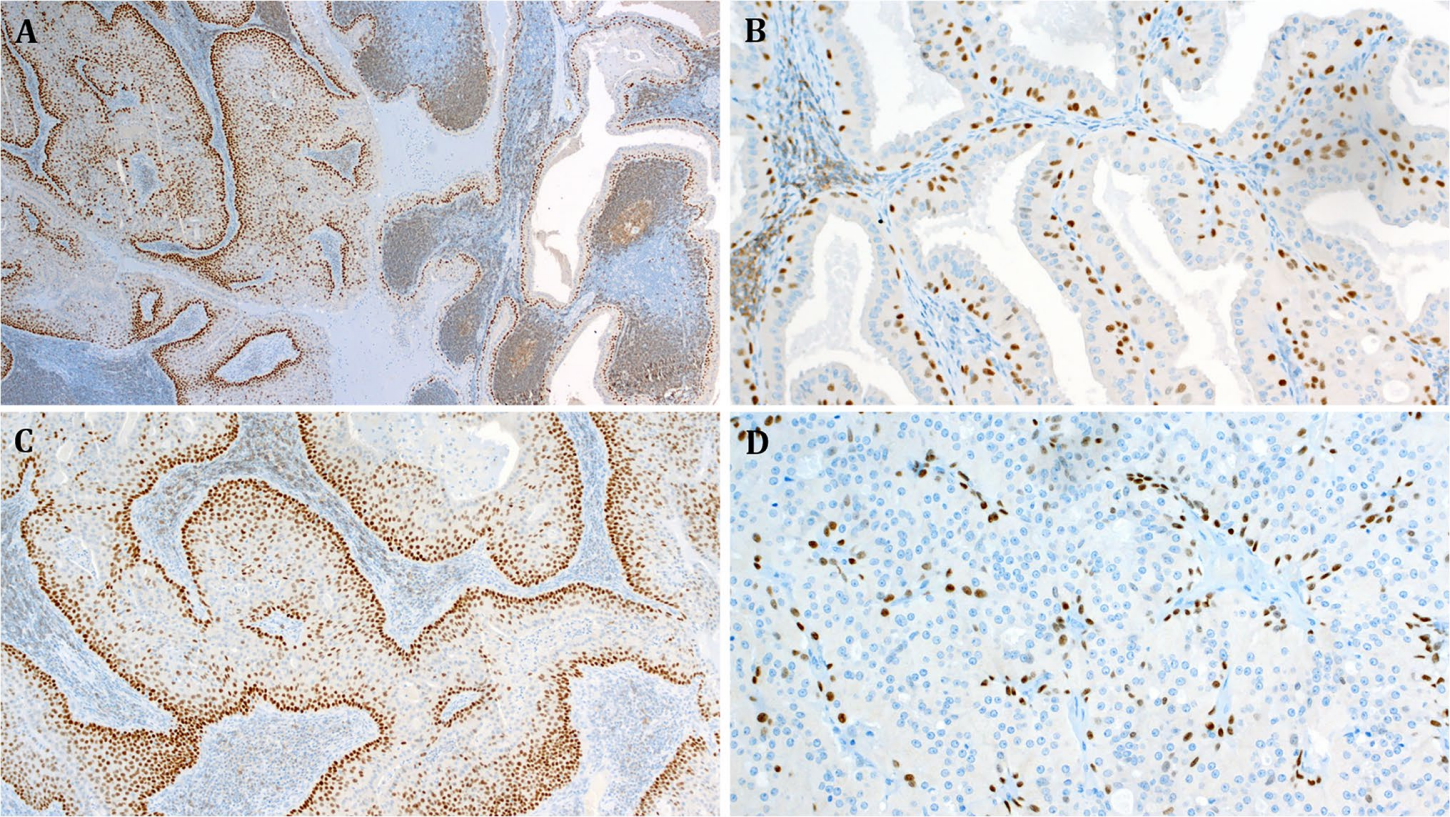
**A** This example of a de novo proliferating Warthin tumor shows transition from conventional component with strictly basal p40 expression (right) to solid epidermoid proliferation with diffuse expression of p40 (left). Higher magnification of the conventional and the proliferating components is illustrated in **B**, **C**, respectively (**A** to **C** case 1). **D** The solid oncocytoma-like component of Case 22 revealed peripheral p40 expression sparing the solid oncocytic aggregates

**Fig. 4:**
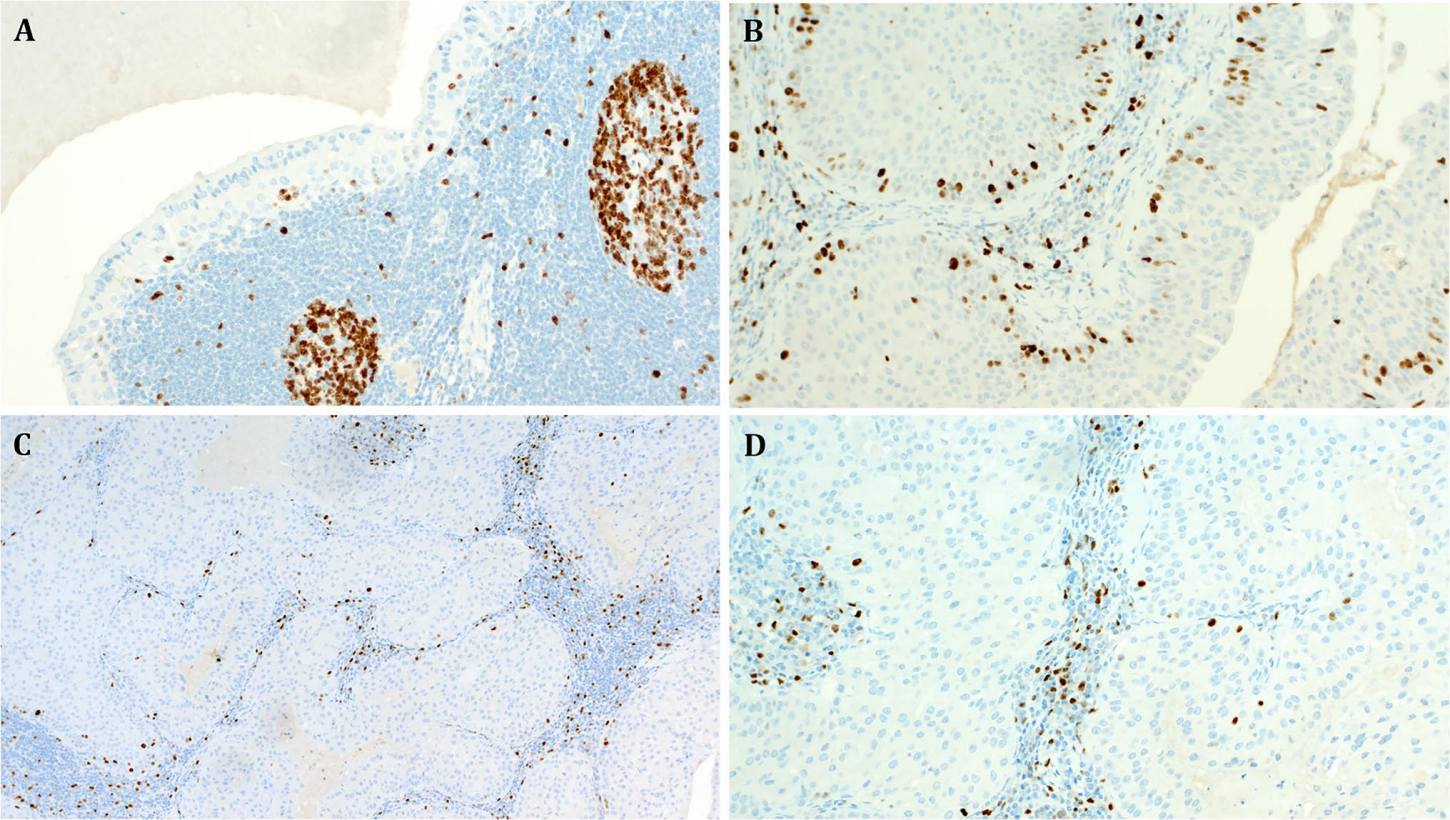
Representative examples of the Ki67 staining pattern in proliferating Warthin tumors (case 17). **A** The conventional component revealed a few Ki67-positive basal cells. **B** Focal increase in the basal proliferation was noted in very few foci at the interphase between the conventional and the proliferating components. **C**, **D** Higher magnifications of the proliferating component of the same tumor showing paradoxically very low Ki67 expression limited to a few basal cells (this is in sharp contrast to what is expected in post-infarction squamous metaplasia)

Immunohistochemistry revealed as expected p40 expression in the atypical solid epidermoid areas, while the conventional areas of the same tumors displayed strictly basal single-cell reactivity for p40 (Fig. 3). The Ki67 highlighted very low single-cell reactivity in the conventional components of proliferating tumors. Remarkably, the solid epidermoid aggregates also lacked increased proliferation in most areas of all rumors except for minor focal increase in suprabasal cells (Fig. 4).

### Molecular findings

**Fig. 5:**
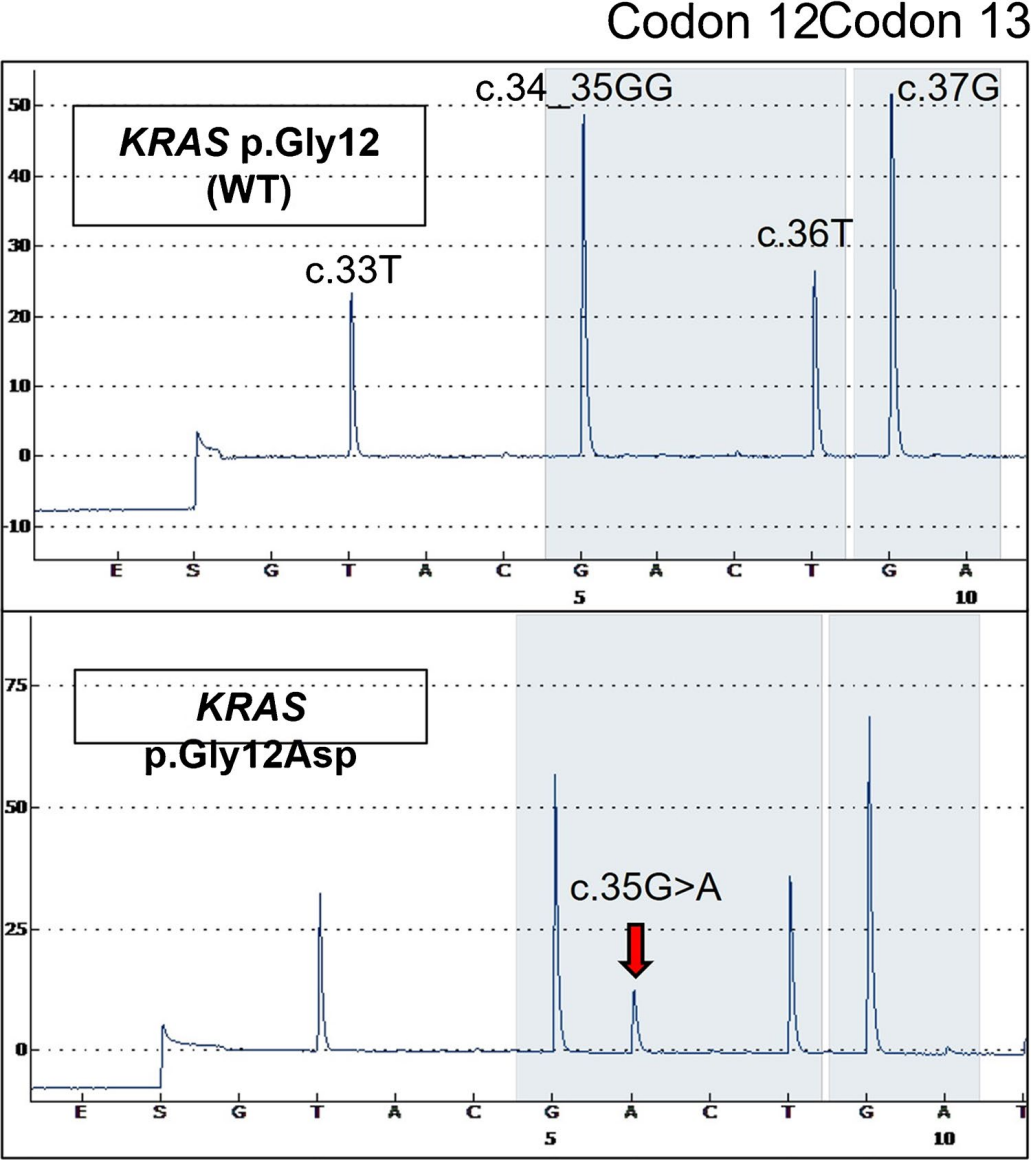
Representative examples of *KRA*S codon 12/13 pyrograms showing the wildtype sequence in the upper lane and the *KRAS* p.Gly12Asp (c.35G > A) mutation in the lower lane (red arrow)

All cases could be successfully evaluated for presence of *KRAS* mutations. A *KRAS* mutation was detected in 6 of the 22 metaplastic tumors (27%), but in none of the 11 conventional WTs in the control group. Three of the mutated cases have been tested by the NGS panel only, while the remainder was tested by both methods; concordant positive and negative results were found in all of the double-tested cohorts. All mutations clustered in codon 12 (exon 2) of *KRAS*. At the amino acid level, they corresponded to the p.Gly12Val (*n* = 3), p.Gly12Asp (*n* = 2), and p.Gly12Ala (*n* = 1) (Fig. 5). The variant allele frequency ranged from 8 to 32%. Notably, of two tumors with the two components tested separately, one revealed a *KRAS* mutation in both components while the other had a mutation restricted to the atypical component. The variant allele frequency was higher for the proliferating tumor component (32%) compared to the conventional mutated component (8%) in the one case with detectable mutation in both components, likely due to the contaminating lymphoid tissue in the latter. Among the proliferating WT cohort, the male to female ratio was balanced for the *KRAS*-mutated (2: 1) and *KRAS*-wildtype (2.2: 1) tumors. *KRAS*-mutated tumors occurred at younger age (57.5 vs. 66) and were slightly larger (median 3.1 vs 2.5 cm) compared to wildtype proliferating tumors (Table 3).

**Table 3:** Compared clinicopathological features in *KRAS*-mutated vs. *KRAS*-wild-type proliferating Warthin tumors

Feature	*KRAS* mutated	*KRAS* wild type
Age range (median)	45–78 (57.5)	50–87 (66)
Male: female ratio	2: 1	2.2: 1
Size range (median) in cm	1.3–3.5 (3.1)	1.5–5.5 (2.5)

All 17 tumors tested for *MAML2* rearrangements by FISH were negative.

## Discussion

Historically considered the second most frequent tumor of the salivary glands after pleomorphic adenoma [1–3], Warthin tumor (WT) represents the most frequent salivary gland tumor in unselected routine cases treated at our center [6, 7]. On the contrary, this tumor seems exceedingly rare in African countries, representing < 1% of all salivary gland lesions in two large series ([8]; Agaimy et al., 2022, unpublished data). Despite its high frequency, the molecular pathogenesis of WT remained elusive and still represents an issue of ongoing controversy [9].

Several studies have failed to detect clonality of the tumor cells, and a non-neoplastic (metaplastic) process involving salivary inclusions within intraparotid lymph nodes has been postulated as a pathogenetic explanation of WTs [10, 11]. The metaplastic theory has been linked to the effect of chronic cigarette smoking, a factor that has also been proposed to explain the striking historical male predominance and the higher frequency of associated head and neck squamous cell carcinoma in patients with WTs [12]. Irrespective of the exact etiology, damage to mitochondrial DNA, possibly resulting from chronic nicotine abuse, and mitochondrial abnormalities including mitochondrial enzyme dysregulations seem to play a role in the morphogenesis of WTs [13, 14]. Currently, there is no general consensus whether WT represents a benign neoplasm or a non-neoplastic metaplastic reactive lesion [1, 11].

The controversy regarding the molecular pathogenesis of WT (especially its metaplastic variant) began decades ago, when cytogenetic studies have shown the presence of the *t*(11;19)(q21;p13.1), later defined as the *CRTC1::MAML2* fusion, in rare WTs [15–17]. However, the findings among these earlier studies were conflicting and inconsistent with some studies showing combinations of normal karyotype, numerical aberrations only, and structural abnormalities, in different subsets of WTs [16–18]. However, none of two recent larger studies could confirm the presence of the *CRTC1::MAML2* or the *CRTC3::MAML2* fusions in any of the conventional or metaplastic WTs analyzed [19, 20]. Using RT-PCR or *MAML2* break apart FISH probes, Skálová et al. could not detect the *CRTC1/3::MAML2* fusion transcripts or *MAML2* rearrangements in any of 16 metaplastic WTs [19]. Another study confirmed the presence of *MAML2* fusion in Warthin-like mucoepidermoid carcinoma, but in none of 114 WTs [20]. However, *MAML2* rearrangements were observed in a subpopulation of cells in the squamous areas of 2 of 8 metaplastic WTs and in 5 of 15 metaplastic WT-like neoplasms in other studies [21, 22]. In one of these studies, the authors were then able to morphologically reclassify all rearrangement-positive cases as WT-like genuine mucoepidermoid carcinomas [22]. Taken together, it seems that *MAML2* rearrangements are exceedingly rare in WTs, and, when present, they likely indicate the presence of concurrent mucoepidermoid carcinoma, namely, the Warthin-like variant of it [20, 22–24].

Parallel to the above-discussed molecular controversy, the precise etiological classification of metaplastic WT and its molecular pathogenesis have been accompanied by a lot of confusion. The major factor responsible for terminological confusion is the vague use of the term “metaplastic” to refer not only to tumors with extensive reparative metaplastic squamous proliferation resulting from tissue injury (due to FNA effect or ischemic-type infarction/necrosis of diverse etiologies [25]), but also to refer to WTs displaying variable proliferating epidermoid or mucoepidermoid component without evidence of preceding tissue injury. Overall, metaplastic changes in WTs fall into two categories: (1) post-FNA or ischemia-induced florid regenerative/reparative metaplastic pseudoepitheliomatous squamous proliferations, frequently closely mimicking squamous cell carcinoma [25], and (2) variable de novo muco-/epidermoid proliferations in native tumors lacking evidence of prior injury. While the former group obviously represents the genuine metaplastic category of WTs and its pathogenesis/etiology is self-explaining, the nature and pathogenesis of the latter (de novo) group remained enigmatic.

To our knowledge, there exists no molecular data on DNA sequence changes (mutations) in WT. Detection of a *KRAS* mutation in a randomly tested index case of WT showing de novo mucoepidermoid-like proliferations but lacking unequivocal mucoepidermoid carcinoma features and lacking detectable *MAML2* rearrangement prompted us to perform the current study to verify the hypothesis, if this mutation is recurrent in this type of WT. We detected a *KRAS* codon 12 mutation in 27% of proliferating WTs, but in none of conventional WTs. Our results shed light on the molecular pathogenesis of de novo variant of proliferating “so called metaplastic” WT. Lack of this mutation in all normal looking WTs is consistent with the notion that the presence of this mutation likely triggers the proliferating component seen in these mutated tumors. Moreover, lack of the mutation in the conventional component of one mutated tumor is also in line with a role for the mutation in driving the mucoepidermoid-like proliferation. Interestingly, a *KRAS* mutation was also detected in the one tumor with an oncocytoma-like proliferating nodule/component, indicating that *KRAS* mutations are not restricted to those tumors with mucoepidermoid-like proliferations but might be found in other atypical-looking cellular variants of WTs as well. However, lack of *KRAS* mutations in the majority of cases (73%) suggests involvement of alternate molecular pathways, possibly affecting genes not included in the small panel we used for this study.

*KRAS* mutations are ubiquitous in benign and malignant tumors across several histological types at different anatomic sites. They represent frequent primary drivers of several common aggressive cancers including the majority of pancreatobiliary carcinomas, colorectal carcinomas, and subsets of non-small cell lung cancer including rare Warthin-like pulmonary adenocarcinoma [26, 27] and subsets of indolent papillary renal cell carcinoma [28]. Moreover, *KRAS* mutations are emerging as major players in a variety of non-neoplastic malformative vascular lesions and benign tumors including subsets of capillary hemangioma [29], non-ossifying fibroma of bone [30], brown tumor of hyperparathyroidism [31] and others.

In contrast to gene fusions, oncogene mutations have been of limited role in benign salivary gland tumors [32]. However, oncogene mutations have emerged recently as potential drivers in several benign salivary gland entities including *BRAFV600E* mutations in sialdenoma papilliferum [33], *AKT1* mutations in intraductal papilloma/ papillary mucinous neoplasms [33, 34], *PIK3CA* mutations in sclerosing polycystic adenoma [35–37], *IDH2* mutations in striated duct adenoma [38], and *HRAS/CTNNB1* mutations in a subset of intercalated duct hyperplasia/adenoma [39]. We herein add a subset of de novo proliferating Warthin tumors to the list of benign salivary gland tumors harboring oncogene (*KRAS*) mutations. *KRAS* mutations are rare in salivary gland tumors and have been mainly detected sporadically in rare malignant neoplasms. In one worldwide meta-analysis study, the overall frequency of *KRAS* mutations in salivary gland tumors was 0.98% compared to a higher (10%) frequency of *HRAS* mutations [40]. However, both mutation types were mostly detected in carcinomas and, only rarely (*HRAS*), in benign lesions.

In summary, we report for the first time oncogenic *KRAS* gene mutations, specific to a subset of de novo proliferating Warthin tumor. Presence of mutations in this variant argues against the “metaplastic theory” and is in line with a neoplastic, albeit benign, lesion. Accordingly, it seems justified to address the two subsets in the historical spectrum of so-called metaplastic WTs separately and to refer to the post-infarction variants as genuine metaplastic WTs, while we propose the descriptive term “de novo proliferating Warthin tumor” for the de novo “metaplastic” variant. While this separation seems currently to carry no prognostic relevance, it would allow for better characterization and better understanding of the molecular background of these tumors. The molecular pathogenesis of the *KRAS*-wildtype de novo proliferating tumors remains an issue of future studies utilizing larger gene panels. Notably, the paradoxically very low proliferative activity in the most striking “proliferating” tumor areas is in line with an abnormal architecture/cellular composition of the tumor rather than a genuine cell proliferation or regenerative metaplasia. Despite this, we feel that the term “proliferating” (in analogy to “proliferating epithelial trichilemmal/epidermoid” cysts [41]) might better reflect and enable specific and reproducible recognition of this morphologically challenging variant of Warthin tumor than the disputed metaplasia terminology.

## Data Availability

The datasets generated during and/or analyzed during the current study are not publicly available, but are available from the corresponding author on reasonable request.
